# QBiC-Pred: quantitative predictions of transcription factor binding changes due to sequence variants

**DOI:** 10.1093/nar/gkz363

**Published:** 2019-05-22

**Authors:** Vincentius Martin, Jingkang Zhao, Ariel Afek, Zachery Mielko, Raluca Gordân

**Affiliations:** 1 Department of Computer Science, Duke University, Durham, NC 27708, USA; 2 Center for Genomic and Computational Biology, Duke University, Durham, NC 27708, USA; 3 Program in Computational Biology and Bioinformatics, Duke University, Durham, NC 27708, USA; 4 Department of Biostatistics and Bioinformatics, Duke University, Durham, NC 27708, USA; 5 Program in Genetics and Genomics, Duke University, Durham, NC 27708, USA; 6 Department of Molecular Genetics and Microbiology, Duke University, Durham, NC 27708, USA

## Abstract

Non-coding genetic variants/mutations can play functional roles in the cell by disrupting regulatory interactions between transcription factors (TFs) and their genomic target sites. For most human TFs, a myriad of DNA-binding models are available and could be used to predict the effects of DNA mutations on TF binding. However, information on the quality of these models is scarce, making it hard to evaluate the statistical significance of predicted binding changes. Here, we present QBiC-Pred, a web server for predicting quantitative TF binding changes due to nucleotide variants. QBiC-Pred uses regression models of TF binding specificity trained on high-throughput *in vitro* data. The training is done using ordinary least squares (OLS), and we leverage distributional results associated with OLS estimation to compute, for each predicted change in TF binding, a *P*-value reflecting our confidence in the predicted effect. We show that OLS models are accurate in predicting the effects of mutations on TF binding *in vitro* and *in vivo*, outperforming widely-used PWM models as well as recently developed deep learning models of specificity. QBiC-Pred takes as input mutation datasets in several formats, and it allows post-processing of the results through a user-friendly web interface. QBiC-Pred is freely available at http://qbic.genome.duke.edu.

## INTRODUCTION

Genetic variants and mutations play important roles in human disease (1). Most variants occur in non-coding genomic regions, where they can impact gene expression by disrupting interactions between transcription factors (TFs) and DNA. In previous work we have introduced an ordinary least squares (OLS)-based method for assessing the impact of non-coding mutations on TF-DNA interactions (2). Briefly, we used high-throughput *in vitro* TF binding data from universal protein-binding microarray (uPBM) experiments (3) to train regression models of TF-DNA binding specificity using OLS estimation. Next, we used the OLS models to predict changes in TF binding due to DNA mutations, and we showed that our binding change predictions correlate well with measured changes in gene expression.

Our approach is novel compared to previous models because, by using OLS, we obtain not only estimates of the model coefficients, but also the variance of these estimates, which allows us to compute normalized binding change scores (*z*-scores) and significance levels (*P*-values) reflecting our confidence that a mutation affects TF binding. The computed *P*-values implicitly take into account the quality of the model and of the training data, so in the case of poor predictive models a large change in binding is required for a mutation to be called significant (2).

Here, we introduce QBiC-Pred (Quantitative Predictions of TF Binding Changes Due to Sequence Variants), or QBiC for short, a web service that allows users to run our OLS models through a user-friendly web interface.

### Input

QBiC takes as input mutation/variant datasets containing single nucleotide variants, in several formats: (i) variant files in the standard variant call format (VCF); (ii) ‘simple somatic mutations’ files generated by the International Cancer Genome Consortium (ICGC) (4); (iii) tab- or comma-separated values files with the columns: chromosome, chromosome_pos, mutated_from and mutated_to; and (iv) text files containing 17-bp DNA sequences with the mutated nucleotide in the center, followed by the ‘mutated to’ nucleotide, separated by a space character. The first three formats can be used with genomic coordinates from versions hg19 and hg38 of the reference human genome, while the sequence format allows users to input custom DNA sequences. For the sequence format, the context of each variant (8-bp on each side) is needed in order to assess the binding status of each allele, using uPBM 8-mer enrichment scores (*E*-scores) (3,5). Examples of input mutation files are described in the ‘About’ section of the website, and available for download. QBiC also takes as input a list of TF proteins of interest, from a list of 582 human TFs with available OLS models. All TF names are specified using the standard HUGO gene nomenclature (HGNC) (6). The list of available TFs and models is available on the QBiC website in the ‘Downloads’ section.

### Output

For each input variant, QBiC runs the OLS models for the list of specified human TFs and it computes the predicted TF binding changes, the normalized changes (*z*-scores), the significance of the changes according to each model (*P*-values), as well as the predicted changes in binding status (e.g. from specific binding, or ‘bound’, to non-specific binding, or ‘unbound’) assessed using uPBM 8-mer data. Similarly to our previous work (7), we consider a site ‘bound’ if it contains two consecutive overlapping 8-mers with *E*-scores > 0.4, and ‘unbound’ is it contains only 8-mers with *E*-score < 0.35; all other sites are called ‘ambiguous’. The *E*-score cutoffs can be modified by the user through the QBiC interface. All computed values are reported as output, in table format. The precise models used by QBiC for each TF protein, as well as the PBM data used to train each model, are reported as part the QBiC results. The user can further process the results using the web interface (e.g. to specify a more stringent *P*-value cutoff for the binding change predictions) and can download the full or filtered results. The web interface also allows users to directly download models or datasets used to obtain individual predictions, and provides links to the HGNC database (6) where users can find additional information about individual TFs.

We are not aware of web servers with the same functionality as QBiC. Users interested in evaluating the putative effects of non-coding mutations on TF-DNA binding can certainly use any of the available databases of position weight matrices (PWMs) (e.g. (8–11)) or deep learning models (12) of TF-DNA binding specificity, or search existing databases of annotations for non-coding variants (e.g. (13,14)). However, such databases do not provide information on the quality of the binding models, and, as shown in the ‘Results’ section below, PWM and deep learning models are not as accurate as our OLS models in predicting the *quantitative* effects of DNA variants on TF binding. The OLS models used in QBiC also have the advantage of providing a direct measure of the significance of each predicted TF binding change, given the model and the training data. This unique feature of our models facilitates interpretation of the results and allows users to prioritize variants for further analysis and validation.

## MATERIALS AND METHODS

### OLS models of TF-DNA binding specificity

The OLS models used by QBiC were trained on curated uPBM data from literature and our laboratory, mapped to 582 human TF proteins. Each uPBM experiment measures the binding specificity of a TF for ∼44 000 60-bp long DNA sequences, each containing a 36-bp variable region followed by a constant 24-bp primer complement (necessary for DNA double-stranding (3)). We use as features the number of occurrences of each possible 6-mer within the 60-bp sequences, and as outcomes the log-transformed fluorescence intensity signals, which reflect the levels of TF binding. The entire 60-bp sequence is used to count 6-mer occurrences, despite the fact that part of the sequence is constant, because the TF proteins can bind at any location within the 60-bp DNA molecule. We consider each 6-mer and its reverse complement as the same variable and combine their counts as one feature, resulting in a total of 2,080 features. The relationship between the outcomes *Y* and the features *X* is modeled by a multiple linear regression *Y* = *Xβ* + *ϵ*.

**Figure 1.**
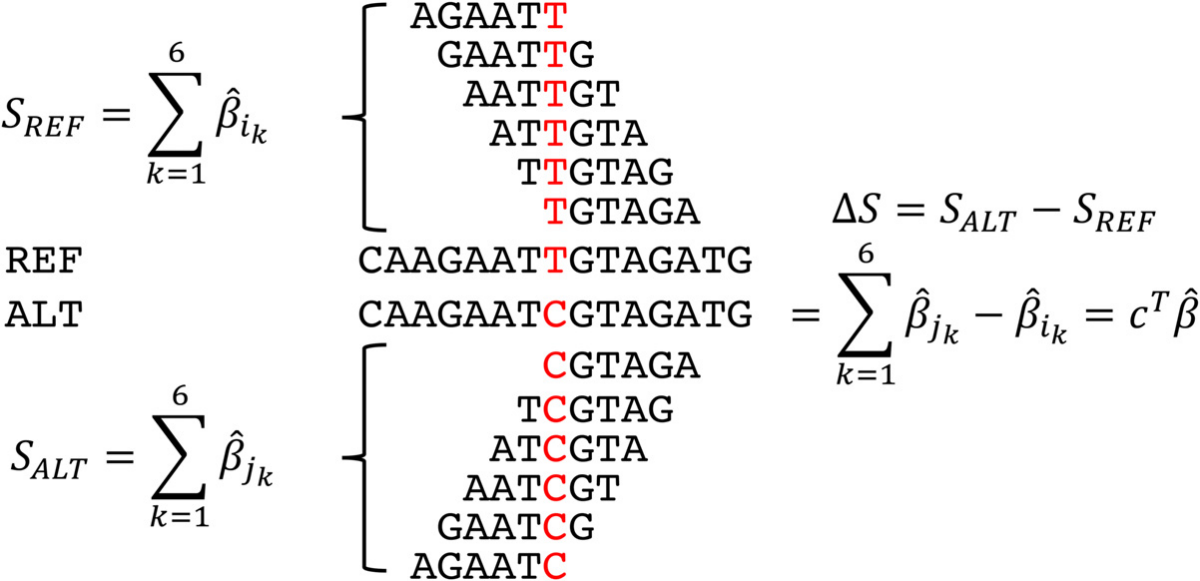
The change in TF binding is computed as a linear combination of the coefficient estimates for all 6-mers overlapping the variant.

To characterize the TF binding change due to a single nucleotide variant, we define binding scores for the wild-type and the mutant sequences, as the sum of the coefficients for all 6-mers overlapping the variant, in an 11-bp window. The difference between these two scores, which represents the binding change, can be expressed as a linear combination of the regression coefficients: *c*^*T*^*β*, where *β* is a vector containing the coefficients for all 2080 6-mer count features, and *c* is a vector of the same length containing, for each 6-mer, the difference in counts due to the variant (Figure 1). We note that most components of *c* are 0, as the variant affects the counts for up to twelve 6-mers.

By further assuming normality on the error term of the linear regression model *ϵ* ∼ *N*(0, σ^2^*I*), we are able to leverage the statistical properties of OLS estimation in order to test whether the binding change is statistically significant. The null hypothesis *H*_0_: *c*^*T*^*β* = 0 can be tested using a t-statistic: $t = {c^T\hat{\beta }}/{\sqrt{c^T\hat{\Sigma }c}}$. Here, $\hat{\beta }$ is the OLS estimate for the coefficients vector: $\hat{\beta }= (X^TX)^{-1}X^TY$, and $\hat{\Sigma }$ is an unbiased estimate for the covariance matrix of $\hat{\beta }$: $\hat{\Sigma }= \hat{\sigma }^2(X^TX)^{-1}$, where $\hat{\sigma }^2 = (Y-X\hat{\beta })^T(Y-X\hat{\beta })/(n-p)$, with *n* being the number of observations and *p* the number of features. Since the regression contains ∼44 000 observations and 2080 variables, this t-statistic follows a t-distribution with ∼42 000 degrees of freedom. Thus, we can use a normal approximation to derive the *z*-score and calculate the *P*-value of the test. For each TF and variant given as input, QBiC calculates and reports the difference in TF binding, the corresponding *z*-score and the associated *P*-value.

To select the uPBM data used in QBiC, we started with 3342 datasets from CIS-BP (10), 245 datasets from UniPROBE (8) that were not included in CIS-BP and 23 datasets generated in our laboratory (7). By using the information in the Human Transcription Factors database (15) for the publicly available uPBM data, and manually curating the data generated in our laboratory, we mapped 1451 uPBM datasets to 638 human TF proteins, using both uPBM experiments that tested human TFs as well as experiments for homologous TFs with high amino-acid identity in the DNA-binding domain region, similarly to Lambert *et al.* (15). Next, to assess the quality of each uPBM data with respect to our task of training accurate quantitative models of TF-DNA binding specificity, we used the cross-validation accuracy of OLS models trained on each uPBM dataset. We removed datasets of poor quality (cross-validation correlation <0.2 computed for the top 10% and top 20% sequences with the highest intensity), and for each TF we selected at most six uPBM datasets, including the top three datasets with the highest cross-validation accuracy, as well as the top three datasets obtained for TFs with the highest amino-acid identify to the human TFs. The final mapping, which includes 667 uPBM datasets and 582 TFs, is available on the QBiC website in the ‘About’ section.

### *In vitro* measurements of TF binding changes due to single nucleotide variants

The PBM technology can be used, with custom-designed DNA libraries, to directly measure the *in vitro* effects of single nucleotide variants on TF binding. To build custom DNA libraries we first selected, at random, DNA sequences containing binding sites for the TFs of interest, and then we introduced all possible single nucleotide variants in the binding site and the immediate flanking regions. Next, we measured the TF binding intensity for all the sequences, and we computed the log ratio of the binding signal between each mutant and the corresponding wild-type sequence to denote the TF binding change due to each variant.

We designed two such DNA libraries and used them to perform custom PBM experiments for six TFs. The DNA library for CREB1, RUNX1 and STAT3 included all single nucleotide variants in the TF binding site (10–12 bp), while the library for ETS1, ELK1 and GATA1 included all single nucleotide variants in the TF binding site and the flanking regions (36 bp). Because several TFs were tested against each DNA library, for each TF we obtain binding data both for variants in their specific binding sites, as well as variants in non-specific regions (which were present in the DNA library because they are specific to other TFs). We used all measurements to evaluate the accuracy of our predictions of TF binding changes (see ‘Results’ section).

### *In vivo* allele-specific binding data

Allele-specific measurements of TF binding from *in vivo* ChIP-seq data have contributed to the identification of genetic variants that have the potential to change TF binding in the cell (16,17). After mapping ChIP-seq reads to each allele of heterozygous variants, allele-specific binding (ASB) events can be identified as the ones with significantly different read counts between the alleles. Here, we used 32 252 ASB events and 79 827 non-ASB events across 81 TFs, as reported in (16), to compare the performance of our OLS-based models versus existing models of TF binding specificity (see ‘Results’ section).

### QBiC-Pred web server implementation

**Figure 2.**
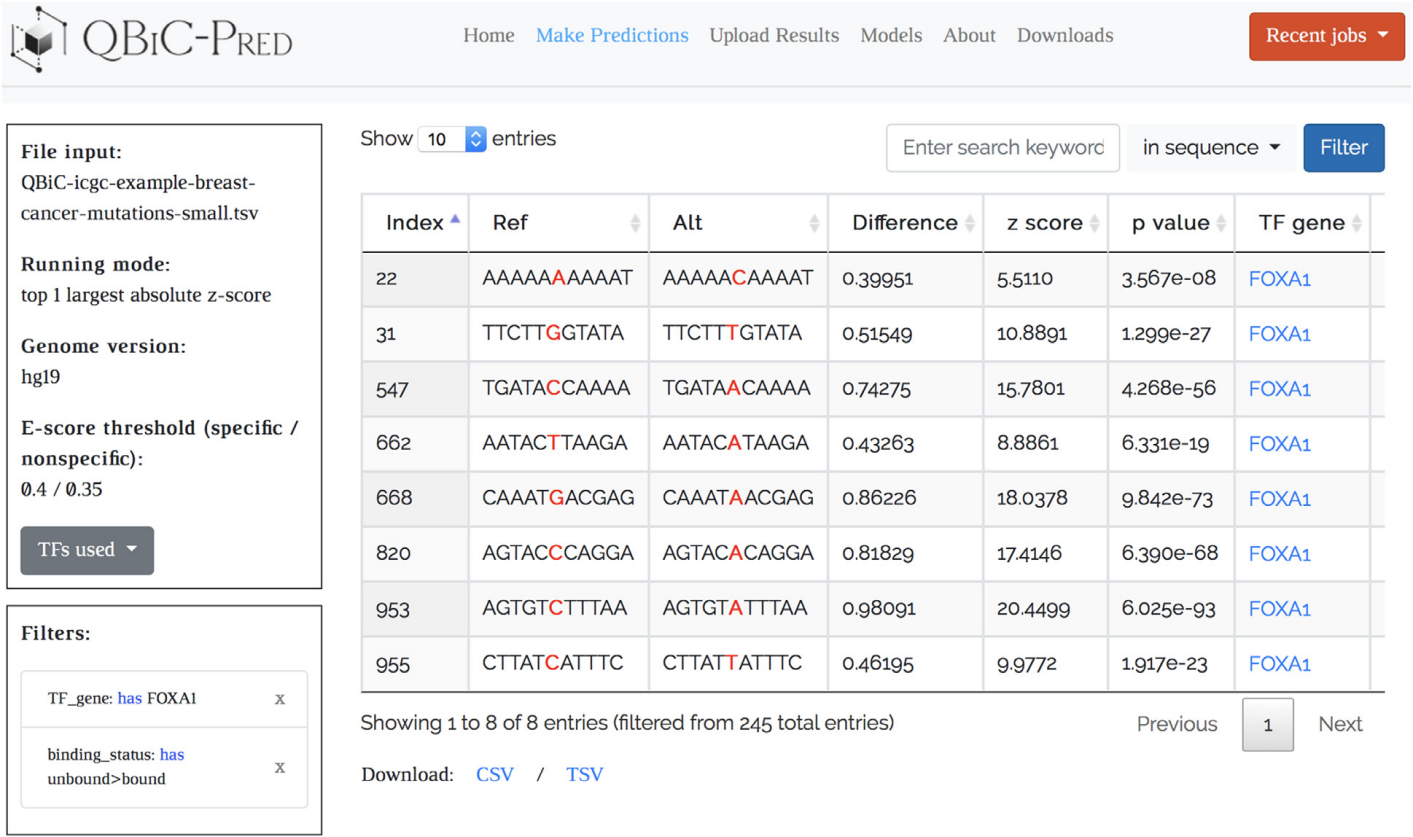
Web server results page for a sample mutation file containing ICGC breast cancer mutation data (the example use case ‘ICGC Breast Cancer Mutations - Small’, available in QBiC). Output results were filtered to include only the FOXA1 transcription factor, and only mutations that create TF binding sites, i.e. ‘unbound>bound’ mutations.

QBiC-Pred was developed using the Flask web framework and it runs under Apache 2.4. Predictions of the effects of input variants on TF binding are made using pre-computed 12-mer tables encoding the predicted TF binding changes, *z*-scores and *P*-values for all possible mutations in all possible contexts (please see the QBiC About page for details). To further speed up the computations, QBiC uses asynchronous multiprocessing with the Celery framework, where four workers (i.e. processes) are spawned for each request. Each worker extracts predictions for a subset of the input TFs. The prediction results are saved in a Redis database for 2 days; during this time the user can access the results using a unique job identifier, and can interactively process the results within QBiC (Figure 2). Users can also download the prediction results and re-upload them later, even after the job expired, for further processing within the QBiC framework.

Users can leave the QBiC website while the predictions are being calculated, and return to the job later using the link provided in the ‘Recent Jobs’ dropdown menu. Importantly, the time needed to execute a prediction job depends mostly on the number of TFs selected as input, as QBiC needs to read into memory the 12-mer table corresponding to each TF. Adding more variants to the input mutation/variant file will have an almost negligible impact on the processing time. After all predictions are computed, they are displayed in a table format with filtering capabilities. Users can post-process the results and download them as csv or tsv files.

## RESULTS

In previous work we showed that our OLS model-based predictions of TF binding changes due to DNA mutations correlate well with measured changes in gene expression (2). We also analyzed a large set of pathogenic non-coding variants, showing that these variants lead to more significant differences in TF binding between alleles, compared to common variants, which indicates that there is a strong regulatory component to pathogenic non-coding variants (2). Here, we complement our previous evaluations of the OLS models by assessing their accuracy in predicting *in vitro* and *in vivo* TF binding changes, and by comparing our OLS models to PWMs and deep learning models of TF binding specificity.

### OLS models of TF binding specificity outperform PWMs and DeepBind models in predicting *in vitro* TF binding changes

As described in ‘Materials and Methods’ section, we designed custom DNA libraries for PBM experiments to test the effects of all single nucleotide variants within binding sites of six human TFs. We used the log ratio of the binding intensity between a mutant and its corresponding wild-type site to represent the TF binding change. Next, we made predictions of these binding changes using six types of models: OLS models, PWM models used in (16), PWM-based sTRAP models (18) and DeepBind models (12) trained on *in vivo* ChIP-seq data, *in vitro* HT-SELEX data and *in vitro* uPBM data. The uPBM datasets used to train DeepBind and OLS models were the same. The PWMs were obtained from the JASPAR (11) and HOCOMOCO (19) databases. For TFs with multiple PWMs available, the results we report below are for the PWM that performed best in our evaluation (ETS1: HOCOMOCO ETS1_HUMAN.H11MO.0.A, ELK1: HOCOMOCO ELK1_HUMAN.H11MO.0.B, GATA1: JASPAR MA0035.2, CREB1: JASPAR MA0018.2, RUNX1: JASPAR MA0002.2, STAT3: HOCOMOCO STAT3_HUMAN.H11MO.0.A). For DeepBind ChIP-seq and SELEX models, we used the v0.11 tools made available for download by the authors (12). For DeepBind PBM models, the authors kindly provided assistance training the models on our uPBM data.

OLS models can directly predict the TF binding change due to a variant in a fixed-length or variable-length sequence. In contrast, for PWM and DeepBind models we computed likelihood scores for the wild-type and mutant sequences, based on fixed-length window scores. For these models, we predicted the binding change as the difference between the maximum of all wild-type window scores and the maximum of all mutant window scores. This definition is the same as delta track metric defined in Wagih *et al.* (16), which performed best in their study.

**Figure 3.**
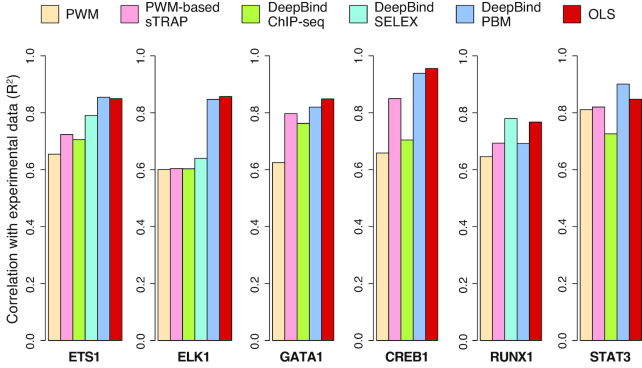
Performance of OLS models in predicting *in vitro* TF binding changes, compared to PWM and DeepBind models. When multiple PWM models are available for a TF, we choose the one that gives the best prediction result. We note that DeepBind ChIP-seq models are not available for RUNX1, and DeepBind SELEX models are not available for GATA1, CREB1 and STAT3. The *in vitro* binding data used in this analysis is available in Supplementary Table S1.

The correlations between model predictions and the TF binding changes measured using custom PBM experiments across the six TFs are shown in Figure 3. Except for RUNX1, for which the DeepBind SELEX model was slightly better than the rest of the models, DeepBind PBM models and our OLS models outperformed the other models in predicting TF binding changes *in vitro*. Compared to DeepBind PBM models, our OLS models are simpler and much faster for training and for predictions. In addition, OLS models can be used to assess the statistical significance of the TF binding changes predicted for each variant.

**Figure 4.**
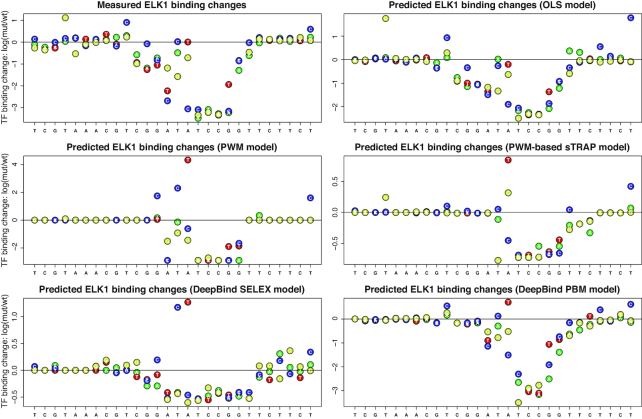
Measured and predicted effects of single nucleotide mutations in an ELK1 binding site and its flanking regions. Since the wild-type sequence contains an ELK1 binding site, most of the variants decrease binding. The A to T mutation in the middle generates a perfect match to the core ELK1 motif TTCC. This, however, does not increase the binding signal, likely because the flanking regions already made the ATCC site very strong. Both the PWM and DeepBind models incorrectly predict a dramatic increase in binding due to the A to T mutation. The OLS model, however, correctly predicts the TF binding to be nearly unchanged. There are also positions where the magnitude of the TF binding change seems to be overestimated by our OLS model but not so much by PWM-based and DeepBind models, such as the T to C mutation at the last position. We note, however, that in this case the correctness of the magnitude of the predicted increase is difficult to assess. For the PWM and the DeepBind SELEX models, the largest predicted increases are incorrect, so we cannot compare them directly to predicted increase at the last position. For the PWM-based sTRAP model and the DeepBind PBM model, the magnitude of the predicted increase at the last position is larger than for other correctly predicted increases, similarly to our OLS model. Thus, it is difficult to judge which model performed best at predicting this particular increase. Nevertheless, over all mutations tested, the OLS model performs best (see also Figure 3).

Figure 4 shows a detailed comparison of five models (OLS, PWM, sTRAP, DeepBind SELEX and DeepBind PBM) for a binding site of TF ELK1. The input mutation file used in QBiC to generate the ELK1 binding change predictions shown in Figure 4 is available as Supplementary Table S2, and can also be downloaded from the QBiC website as the sample input file in sequence format.

### The cross-validation accuracy of OLS models correlates with their accuracy in predicting *in vitro* TF binding changes

**Figure 5.**
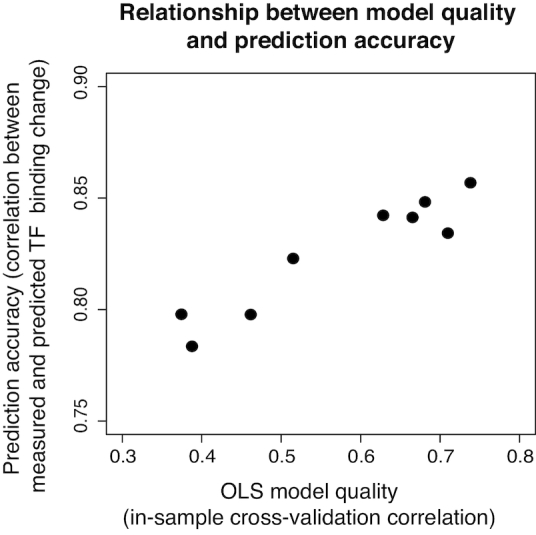
Relationship between OLS model quality (assessed as the in-sample cross-validation correlation) and the prediction accuracy on independent *in vitro* mutation data. Figure shows the performance of OLS models trained on nine different uPBM datasets for TF ELK1.

A TF can have multiple PWM models and DeepBind models available, and it is often difficult to choose which model to use for prediction. In contrast, for our OLS-based approach, we are able to rank the models based on cross-validation accuracy on the uPBM training dataset. As expected, we found that there is a positive relationship between the in-sample cross-validation accuracy and the TF binding change prediction accuracy on independent *in vitro* data (Figure 5). Thus, when a TF has multiple OLS models, we recommend choosing the model with the highest cross-validation accuracy. Detailed information on the available OLS models for each human TF can be found in the ‘About’ section of the QBiC website.

### OLS models of TF binding specificity outperform PWMs and DeepBind models in predicting *in vivo* allele-specific binding variants

To test the performance of OLS models on *in vivo* data, we used the allele-specific binding (ASB) and non-ASB variants in (16). We compared the performance of OLS models, PWM models and DeepBind models in distinguishing ASB variants from non-ASB variants. The performance of each model was assessed using the area under the Receiver Operating Characteristic curve (AUROC) measure. For PWMs and DeepBind ChIP-seq models, we used the binding change scores reported by Wagih *et al.* (16). For DeepBind SELEX and PBM models we derived the binding change scores similarly to Wagih *et al.* (16), and used them for the classification. For OLS models we used the *z*-score outputs to classify the variants. The DeepBind PBM and OLS models were trained on the same sets of PBM data. To illustrate how QBiC can be used to analyze ASB and non-ASB variants, in Supplementary Table S3 we provide the input mutation file corresponding to the ASB data for TF MAFK, in VCF format. This file is also available on the QBiC website, as the sample input file for the VCF format.

**Figure 6.**
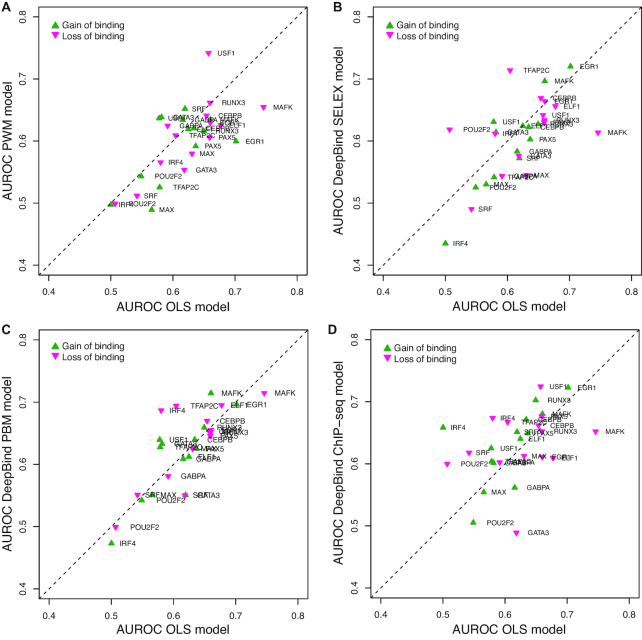
Performance of OLS, DeepBind and PWM models in distinguishing between ASB and non-ASB variants identified from *in vivo* ChIP-seq data.

A total of 14 human TFs have PWM models, OLS models, and DeepBind models available. For these TFs we divided their ASB variants into gain-of-binding and loss-of-binding variants (for which the TF binding changes have opposite signs), and for each set we used the different TF binding models to distinguish between ASB and non-ASB variants. OLS models clearly outperformed PWMs (Figure 6A), which was expected given the limitations of PWM models in capturing TF binding specificity (7,20–22). OLS models also outperformed DeepBind SELEX models trained on *in vitro* binding data from HT-SELEX experiments (Figure 6B) and DeepBind PBM models trained on *in vitro* data from PBM experiments (Figure 6C) demonstrating that, when using only DNA sequence information for training, OLS models perform best in predicting *in vivo* allele-specific binding variants.

We also compared the performance of OLS models to DeepBind models trained on *in vivo* ChIP-seq data (Figure 6D). Using OLS models we obtained larger AUROC values for about half of the TFs, and overall the two models had similar power in distinguishing ASB from non-ASB variants. Nevertheless, we note that the DeepBind ChIP-seq models were trained on ChIP-seq data from the same cell type as the ChIP-seq data from which the ASB variants were called. Therefore, OLS models managed to reach similar performance to models trained on the ChIP-seq data itself, despite the fact that OLS models do not use any cell type-specific information.

## DISCUSSION

Quantitative predictions of TF binding changes can help us understand the functional roles of genetic variants, and prioritize variants that are likely to have regulatory effects. QBiC-Pred provides a fast and accurate approach to predict TF binding changes due to genetic variants, based solely on their sequence context. QBiC-Pred models are trained on *in vitro* high-throughput universal PBM data, and they outperform current PWM-based models and DeepBind models, which are also based mainly on DNA sequence information. In addition, QBiC-Pred offers a way to statistically test the significance of each variant, taking the quality of the predictive models into account. The quality measure of the models also helps circumvent the problem of deciding which model to use when multiple models are available, which is often encountered when making predictions using PWMs.

Several recent methods, including Sasquatch (23), DeepSEA (24) and deltaSVM (25), predict the impact of non-coding variants by taking advantage of cell- and tissue-specific information, oftentimes beyond TF binding data. These methods are complementary to ours: they focus on overall functional changes caused by non-coding variants, while we examine more specifically the potential binding changes for each individual TF. For example, Sasquatch predicts the change in the DNase footprint due to a variant, but does not directly pinpoint the binding of which TF(s) is affected by the variant (unless one post-processes the results using specific TF binding models). In contrast, QBiC-Pred can make quantitative predictions in a TF-specific manner, for a large number of TFs, although it cannot predict the effect of the variant in any specific cell type. Using these methods together would give us a better understanding of the functional impact of non-coding variants in the cell.

Annotation-based methods such as rVarBase (26), INFERNO (27), HaploReg (28) and RegulomeDB (14) can also be used to investigate potential regulatory variants. These methods test whether the input variants fall within known regulatory regions annotated, for example, using PWM models and cell type-specific data. Thus, predictions made by annotation-based methods depend on the quality of the existing annotations, and, in the case of TF binding sites, these methods are unlikely to detect variants that lead to the creation of new binding sites in the genome. In addition, we note that none of the methods mentioned above provides a direct measure of the confidence in the predicted changes in TF binding, based on the quality of the binding data and model, which is a distinguishing feature of QBiC-Pred.

In summary, QBiC-Pred uses OLS models of TF-DNA binding specificity to make accurate predictions of TF binding changes due to single nucleotide variants. In addition to the current functionalities of QBiC-Pred, a natural extension would be to allow input sequences containing multiple variants. As shown in our previous work, OLS models perform very well on data containing multiple variants, being able to predict ∼50% of the resulting variation in gene expression (2). Another extension would be to include models trained on other types of high-throughput *in vitro* TF binding data, such as HT-SELEX data (29,30). This would extend the list of human TFs that can be analyzed using QBiC-Pred beyond the 582 TFs with available high-quality uPBM data. This extension, however, will require the development of new methodology that takes into account the statistical properties of the HT-SELEX data, in order to allow us to use the data directly to compute significance levels (*P*-values) reflecting our confidence in the predicted effects of mutations on TF binding.

## DATA AVAILABILITY

All the raw and process PBM data generated and used in this study are available in GEO (SuperSeries GSE130837). The processed data are available in Supplementary Table S1.

## Supplementary Material

gkz363_Supplemental_Files
